# Co-contraction patterns of trans-tibial amputee ankle and knee musculature during gait

**DOI:** 10.1186/1743-0003-9-29

**Published:** 2012-05-28

**Authors:** Mahyo Seyedali, Joseph M Czerniecki, David C Morgenroth, Michael E Hahn

**Affiliations:** 1 Department of Veterans Affairs (VA), Rehabilitation Research and Development Center of Excellence for Limb Loss Prevention and Prosthetic Engineering, VA Puget Sound, 1660 S. Columbian Way, Seattle, WA 98108, USA; 2 University of Washington, Department of Mechanical Engineering, 4518 University Way Northeast, Seattle, WA 98105, USA; 3 University of Washington, Department of Rehabilitation Medicine, 1959 NE Pacific Street, Box 356490, Seattle, WA 98195, USA

**Keywords:** Electromyography, Myoelectric control, Trans-tibial amputees, Activation patterns, Muscle amplitude

## Abstract

**Background:**

Myoelectric control of upper extremity powered prostheses has been used clinically for many years, however this approach has not been fully developed for lower extremity prosthetic devices. With the advent of powered lower extremity prosthetic components, the potential role of myoelectric control systems is of increasing importance. An understanding of muscle activation patterns and their relationship to functional ambulation is a vital step in the future development of myoelectric control. Unusual knee muscle co-contractions have been reported in both limbs of trans-tibial amputees. It is currently unknown what differences exist in co-contraction between trans-tibial amputees and controls. This study compares the activation and co-contraction patterns of the ankle and knee musculature of trans-tibial amputees (intact and residual limbs), and able-bodied control subjects during three speeds of gait. It was hypothesized that residual limbs would have greater ankle muscle co-contraction than intact and able-bodied control limbs and that knee muscle co-contraction would be different among all limbs. Lastly it was hypothesized that the extent of muscle co-contraction would increase with walking speed.

**Methods:**

Nine unilateral traumatic trans-tibial amputees and five matched controls participated. Surface electromyography recorded activation from the Tibialis Anterior, Medial Gastrocnemius, Vastus Lateralis and Biceps Femoris of the residual, intact and control limbs. A series of filters were applied to the signal to obtain a linear envelope of the activation patterns. A co-contraction area (ratio of the integrated agonist and antagonist activity) was calculated during specific phases of gait.

**Results:**

Co-contraction of the ankle muscles was greater in the residual limb than in the intact and control limbs during all phases of gait. Knee muscle co-contraction was greater in the residual limb than in the control limb during all phases of gait.

**Conclusion:**

Co-contractions may represent a limb stiffening strategy to enhance stability during phases of initial foot-contact and single limb support. These strategies may be functionally necessary for amputee gait; however, the presence of co-contractions could confound future development of myoelectric controls and should thus be accounted for.

## Background

Myoelectric control of upper extremity powered prostheses has been used clinically for many years. Though not yet fully developed, electromyography (EMG) inputs may soon be used to assist in the control of powered prosthetic devices for lower limb amputees. Myoelectric control offers potential advantages such as user intent input through activating certain muscles to control different locomotion states or to provide proportional online control. Before development of myoelectric control for lower extremity prostheses occurs, it is necessary to first understand the myoelectric characteristics of amputee residual and intact musculature and to analyze unusual co-contraction patterns that may arise.

The EMG signals from individual lower limb muscles throughout the gait cycle are generally consistent across subjects during able-bodied walking [1-6]. Although myoelectric activity of trans-tibial amputees might be expected to differ from that of able-bodied individuals because of the differences in joint kinetics [7-9], some groups have reported similar EMG patterns of the residual limb knee musculature compared to those of the intact limb and control limbs of able-bodied individuals [8,10,11]. However, a few studies have highlighted key differences in patterns between limbs. Specifically, residual limb knee flexor muscles have been shown to exhibit increased activation from initial contact to midstance [7,10,12], with longer durations [7,8] and delayed peaks [12]. Intact limb knee flexor muscles are equally active in early stance and late swing. However, residual limb knee flexors have been shown to be four times more active in early stance compared to late swing [12]. Residual limb knee extensors have also been reported to demonstrate increased activation and duration through heel strike, but not to the same extent as the knee flexor group [7,8,10,12]. Although these reports indicate that increased co-contraction may exist in the knee musculature of trans-tibial amputees, other studies have reported a lack of knee muscle co-contraction in trans-tibial amputees [13,14]. From these studies, it is apparent that co-contraction patterns in the knee musculature of trans-tibial amputees differ from controls; however the exact mechanism or reason for that difference is not well understood.

Regarding EMG activation patterns of the residual limb ankle musculature in trans-tibial amputees, Au and colleagues reported that in a stationary (non-walking) case, an amputee’s residual limb ankle muscle signals are capable of producing signals that reflect the individual’s intent [15]. Furthermore, EMG signals in the residual limb ankle muscles of trans-tibial amputees have been used as an on/off switch for different control states of an experimental prosthetic device [16]. Although minimal research has been reported regarding in-socket EMG in trans-tibial amputees, a few studies have reported in-socket EMG patterns of trans-femoral amputees. Recognition of user intent has been demonstrated in trans-femoral amputee gait, with some muscles exhibiting very distinct activation patterns during gait transitions [17,18]. From these observations, Huang et al. concluded that it is possible to discriminate between different modes of walking (level, ramp ascent and descent) from the EMG signals of residual and intact limb muscles [18].

It has become apparent that amputees retain neural response capabilities in their residual musculature but it is unknown to what extent, and how their myoelectric patterns compare with those of controls. Initial analysis of preliminary trans-tibial amputee data revealed unusual co-contraction patterns in the residual ankle musculature during phases of transition within a gait cycle. Damiano et al. postulated that co-contraction of residual musculature may be the result of a protective stabilization mechanism [19], however this notion needs to be investigated further with respect to trans-tibial amputees to augment our understanding of the myoelectric characteristics of the residual muscles.

Due to increased mechanical demands associated with increased walking speed, it is reasonable to expect activation of the lower extremity muscles to increase with speed of gait [1,5,20]. There is limited understanding however regarding how speed may affect relative changes between residual limb myoelectric signals in amputees. The activation of knee muscles in the residual and intact limbs of trans-tibial amputees has been reported to increase in magnitude with added speed demands [10,21]. However, it remains unknown whether increased activation of residual limb agonist and antagonist muscles is relatively equal or not, directly influencing co-contraction levels. It is important to assess which demands (e.g. speed, certain phases of gait) contribute to unusual co-contractions in amputees to better understand the natural function of muscle activity in amputees.

Although the in-socket muscles of the residual limb apparently retain activation patterns, the patterns do not necessarily follow those of able-bodied individuals [18]. It is also evident that the more proximal, out-of-socket muscles exhibit different co-contraction patterns when compared to able-bodied controls [7,8,11,13,14]. Furthermore, it is currently unknown what differences exist between the co-contractions of residual and intact limb ankle musculature (in-socket) and knee musculature (out-of-socket) in trans-tibial amputees, when compared to those of controls, and what effect walking speed may have on co-contraction levels. An increased understanding of these differences will further expand our knowledge regarding the characteristics of amputee myoelectric patterns. This may help guide the development of future myoelectric controllers, as any co-contraction patterns can thus be accounted for.

The purpose of this study was to analyze activation and co-contraction patterns of ankle and knee agonist/antagonist muscle pairs, specifically the Tibialis Anterior (TA), Medial Gastrocnemius (MG), Vastus Lateralis (VL) and Biceps Femoris (BF) during three speeds of gait. Three hypotheses were tested in this study. First, it was hypothesized that residual limbs would have greater co-contraction of the TA and MG muscles than intact and control limbs due to the need for enhanced stability arising from the increased instability inherent to walking with a prosthetic limb. The second hypothesis was that co-contraction of the VL and BF would be different between all limbs. Lastly, it was hypothesized that co-contraction levels would increase with walking speed.

## Methods

Fourteen male individuals participated in this study, nine of which were unilateral trans-tibial amputees (50 ± 14 years; 1.81 ± 0.06 m; 86 ± 14 kg). The remaining five were matched control subjects (50 ± 15 years; 1.83 ± 0.04 m; 87 ± 7 kg). All trans-tibial amputee subjects walked on their prescribed prosthetic components (seven of which were Flex-Foot®; Ossur, Reykjavik, Iceland). All subjects’ prosthetic feet consisted of passive energy storage and return devices (average mass: 729 ± 364 g; range 310–1150 g). Seven subjects used pin lock suspension systems, while two used anatomic suspension and suction mechanisms. The protocol was approved by the associated Institutional Review Boards. Written informed consent was obtained prior to each subject’s participation.

**Figure 1:**
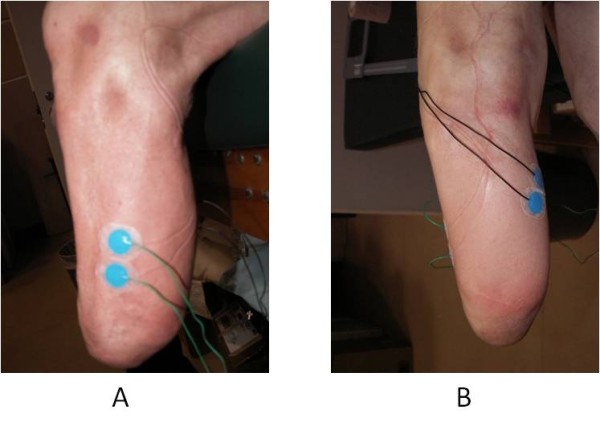
**Example of bipolar electrode configuration.** Bipolar electrode configuration displayed on a sample subject. The electrodes were neonatal disposable wet-gel Ag/AgCl passive electrodes. (**A**) Lateral view of the residual limb with electrodes placed on the Tibialis Anterior. (**B**) Posterior-lateral view of the residual limb with electrodes placed on the Medial Gastrocnemius.

**Figure 2:**
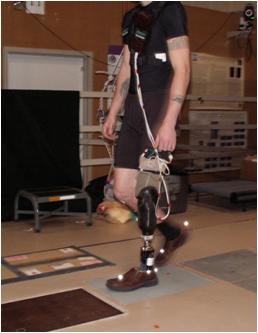
**Example of system setup and walking condition.** The EMG gain box is shown strapped to the subject’s chest. After signal gain and band-pass filtering, the EMG signals were transmitted via FM radio frequency to a receiver near the analog breakout box and A/D board.

Surface EMG recorded muscle activation at 1200 Hz from the TA, MG, VL and BF bilaterally using disposable wet-gel neonatal passive electrodes in a bi-polar single differential configuration (Figure 1). These muscles were selected based on previous amputee knee muscle literature [2,7,8,11,12], and an assessment of the relative muscle mass remaining in the residual limb. In addition, the selected muscles represent the primary muscle groups and agonist/antagonist pairs of the specific joint and are easily accessible to ensure reliability of EMG sensor placement. Although prosthetic motion on surface electrodes may provide additional noise to the signal, these low-profile electrodes were tested in a pilot study to reveal minimal differences upon shear testing. The main source of noise in the signal was from sensor cable motion between the socket and the amplifiers. Cable-motion artifact is easily detected and filtered because of its low frequency and high amplitude characteristics. For the intact and control limbs, electrode placement was based on Delagi and Perotto’s standards [22]. For the residual limb, the same standards were used as a guideline with actual placement adjusted minimally according to thorough palpation and signal confirmation. Foot marker trajectories (collected with a 12-camera Vicon MX system at 120 Hz; Oxford, UK) in combination with ground reaction force data (collected with 2 AMTI, 2 Bertec, and 1 Kistler force platform at 1200 Hz) were used to determine gait cycle events. Subjects were asked to walk over a 10 m walkway (Figure 2), with five trials collected at three different speeds; self-selected walking speed (SSWS), 10% slower than SSWS, and 10% faster. The subject’s SSWS was determined using a hallway walking test where the subject was timed as they walked at their comfortable, normal pace for a known length of 19.63 meters.

**Figure 3:**
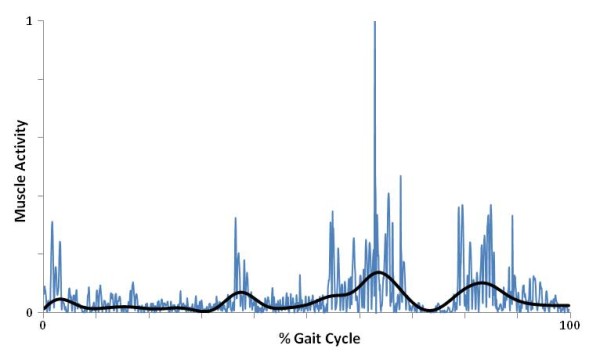
**Example of raw data overlaid with a linear envelope.** Myoelectric activity of the Tibialis Anterior is shown for one gait cycle. The raw signal seen in the blue line represents rectified myoelectric activity that has been initially filtered through the hardware band-pass filter (10-500 Hz) and full-wave rectified. The overlaid signal in black represents the linear envelope signal, filtered through the following steps: (**1**) High pass filter (50 Hz) to remove motion artifact, (**2**) Notch filter (59.5-60.5) to remove ambient power line noise, (**3**) Full-Wave Rectification, (**4**) Low pass filter (8 Hz) to smooth the final signal.

Raw EMG signals recorded from all walking trials were processed using a sequence of analog and digital filters. The EMG collection hardware (Telemyo, Noraxon, Scottsdale, AZ) provided a signal gain of 5,000 and a band-pass filter of 10–500 Hz to remove known non-muscle frequencies. The remaining signal was filtered using custom written Matlab code (Mathworks, Natick, MA) through the following sequence. First, a 4th order Butterworth high pass filter with a cut off value of 50 Hz was used to remove motion artifact from cable motion. The cut off value of 50 Hz was chosen after conducting preliminary post-processing tests where different cut off values were applied to the signal to optimally remove the most motion artifact while preserving the most muscle signal. Next, a notch filter was applied with cut off values of 59.5 and 60.5 Hz to remove ambient power line noise at 60 Hz. Following full wave rectification, a linear envelope was created using a 4^th^ order Butterworth low pass filter with a cut off value of 8 Hz. Lastly, the linear envelope was amplitude normalized to the largest value of each subject’s EMG during fast walking and time normalized to 100% gait cycle (Figure 3).

Co-contraction analysis was separated into two functional activation categories, ankle dorsi- and plantar flexors (represented by TA and MG), and knee extensors and flexors (represented by VL and BF). Analysis of ankle muscle activity involved three phases of gait: early stance (0-10% gait cycle), mid-late stance (20-60% gait cycle) and early swing (60-80% gait cycle). These phases of gait were chosen due to the presence of unusual co-contractions observed during initial pilot analysis. The amount of co-contraction was quantified using a co-contraction area (CCA) calculation adapted from Damiano et. al. (2000)[23]; a ratio between integrated antagonist and agonist signals during each designated phase of gait.

(1) $$CCA=\frac{\int Antagonist}{\int Agonist}$$

Table 1 lists agonist and antagonist designations for each phase of gait based on able-bodied walking. Applying this standard, during early stance (0-10% gait cycle), the agonist muscle group was considered to be the dorsiflexors (represented by TA) as they eccentrically control the plantar flexion that occurs from heel-strike to foot-flat. During mid-late stance (20-60% gait cycle) the agonist was designated as the plantar flexor group (represented by MG), due to the eccentric control of tibial progression and the concentric initiation of push off. During early swing (60-80% gait cycle) the agonist was considered to be the dorsiflexor group (TA), as they control the position of the foot during the swing phase of gait. Previous research has demonstrated that in normal conditions there should be minimal co-contraction in these phases due to the dominant patterns of the agonist muscle groups [1-5].

**Table 1:** Ag/Antagonist designations for the ankle and knee

**Gait cycle phase**	**Agonist**	**Antagonist**
Early Stance (0-10%)	TA	MG
Early-Midstance (0-20%)	VL	BF
Mid-Late Stance (20-60%)	MG	TA
Early Swing (60-80%)	TA	MG
Late Swing (80-100%)	BF	VL

Analysis of knee muscle activity involved two phases of gait; early-midstance (0-20% gait cycle) and late swing (80-100% gait cycle). These specific phases were chosen due to previous reports of prevalent co-contractions within each phase [2,8,9,12,13,18]. During normal gait both the VL and BF are activated in these phases to prepare for heel-strike and control of the leg as foot-flat occurs. However, each phase consists of one muscle group activating more than the other to control the movement, therefore that group was designated as the agonist. During early-midstance (0-20% gait cycle), the dominant muscle group was designated to be the knee extensors (represented by VL), as they eccentrically stabilize the knee from heel-strike through foot-flat. During late swing (80-100% gait cycle) the dominant muscle group was designated to be the knee flexors (represented by BF), as they eccentrically decelerate hip flexion and knee extension to prepare for heel-strike [1-4,6,24].

A two-factor ANOVA (limb, walking speed) with repeated measures on walking speed was used to test each hypothesis (α = 0.05). An ANOVA was run for each phase of gait studied; therefore three ANOVAs were used for the ankle results and two for the knee results. For specific limb-to-limb comparisons during each phase, post-hoc analysis consisted of a two-sample *t*-test with Bonferroni adjustment for multiple comparisons. All statistical tests were conducted using Systat (v. 12, Systat Software, Inc., Chicago, IL).

## Results

Fourteen individuals participated in this study and were split into two groups, an amputee group and a control group. There were no significant demographic or speed effects between the two groups (see Table 2). For all muscle co-contraction analyses, ten sets of limb-specific data were included in the control ensemble average, using both limbs of the five control subjects. Nine sets of limb-specific data (from the nine amputee subjects) were included in both the intact and residual limb ensemble averages.

**Table 2:** Demographics and walking speeds for both groups

	**Trans-tibial**	**Controls**	***p*-value**
	Demographics		
Age	50 ±14 years	50 ± 15 years	0.74
Height	1.82 ± 0.06 m	1.83 ± 0.04 m	0.64
Weight	86.4 ±13.9 kg	87.3 ± 7.3 kg	0.88
	Speeds (m/s)		
Slow	1.02 ± 0.05	1.08 ± 0.15	0.21
SSWS	1.25 ± 0.07	1.20 ± 0.15	0.34
Fast	1.44 ± 0.14	1.33 ± 0.18	0.64

**Figure 4:**
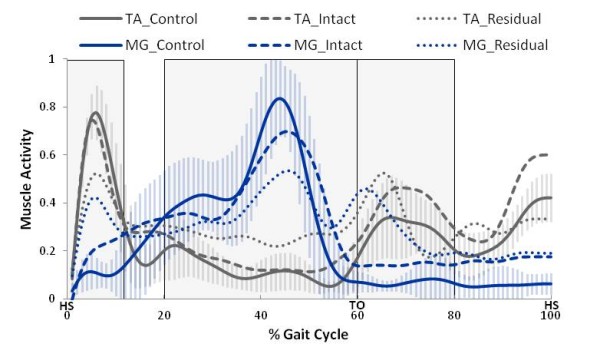
**All limbs ankle muscle activation patterns across one SSWS gait cycle.** Ensemble average ankle muscle activation patterns for all limbs across gait cycle are shown for self-selected walking speeds. Muscle activity was normalized to the maximum activation recorded during fast walking. The shaded areas represent the phases of gait examined. Heel strike (HS) and toe off (TO) events are labeled in the horizontal axis. The vertical bars represent one standard deviation for the control limb. The residual limb had the most variation with an average standard deviation of 0.175 for the TA and 0.180 for the MG followed by the intact and control limbs. The intact limb average standard deviation was 0.133 for the TA and 0.149 for the MG. The control limb average standard deviation was 0.097 for the TA and 0.119 for the MG.

Muscle activation patterns of the trans-tibial intact limb were qualitatively very similar to those of the control limb. However, the activation patterns of the residual limb showed greater variation in the ankle and knee muscles. Figure 4 displays the ensemble average activation patterns for the control, intact and residual limbs’ ankle musculature. There was no significant effect of walking speed on any of the co-contraction measures. Therefore, only self-selected speed activation patterns are displayed.

Co-contraction levels for the ankle muscles were significantly different between limbs during each of the three phases of gait (see Table 3). Specifically, during early stance, mid-late stance and early swing, the residual limb ankle muscle CCA was greater than the intact (*p* < 0.001) and control limb (*p* < 0.001), and the intact limb ankle muscle CCA was also greater than the control limb (*p* < 0.002). There was no significant speed effect on ankle muscle CCA values during any phase of gait (*p* > 0.322).

**Table 3:** CCA values for the ankle muscles during three speeds of gait; Mean (SD)

	**Gait cycle phase**								
	Early stance (0-10 %)^*^			Mid-late stance (20-60 %)^*^			Early swing (60-80 %)^*^		
**Speed**	**R**^**a**^	**I**	**C**	**R**	**I**	**C**	**R**	**I**	**C**
Slow	0.98 (0.90)	0.37 (0.31)	0.15 (0.07)	1.0 (0.69)	0.52 (0.34)	0.29 (0.10)	1.1 (0.66)	0.46 (0.38)	0.24 (0.17)
SSWS	1.0 (1.0)	0.40 (0.33)	0.16 (0.11)	1.0 (0.89)	0.50 (0.32)	0.31 (0.12)	0.93 (0.45)	0.43 (0.28)	0.24 (0.19)
Fast	1.2 (0.73)	0.33 (0.26)	0.15 (0.10)	0.88 (0.36)	0.43 (0.18)	0.29 (0.11)	0.85 (0.31)	0.43 (0.30)	0.23 (0.18)

^**a**^ R = Residual, I = Intact, C = Control; ^*^ Significant limb effect for all phases in the ankle (*p* < 0.02).

**Figure 5:**
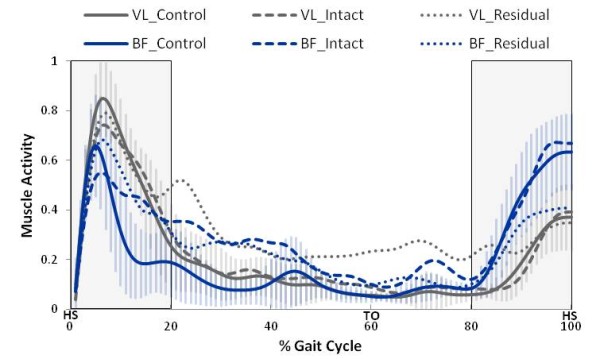
**All limbs knee muscle activation patterns across one SSWS gait cycle.** Ensemble average knee muscle activation patterns for all limbs across gait cycle are shown for self-selected walking speeds. Muscle activity was normalized to the maximum activation recorded during fast walking. The shaded areas represent the phases of gait examined. The vertical bars represent one standard deviation for the control limb. Heel strike (HS) and toe off (TO) events are labeled in the horizontal axis. Overall, there was less variance in the knee musculature compared to the ankle musculature. The residual limb average standard deviation was 0.153 for the VL and 0.131 for the BF. The intact limb average standard deviation was 0.090 for the VL and 0.143 for the BF. The control limb average standard deviation was 0.086 for the VL and 0.102 for the BF.

Figure 5 displays the ensemble average activation patterns of the knee muscles for the control, intact and residual limbs’ knee musculature during self-selected walking speed. During early-midstance and late swing there was not a significant overall limb effect for co-contraction levels, however there were specific inter-limb differences (see Table 4). During early-midstance the residual limb knee muscle CCA was not different than the intact limb (*p* = 0.114) but was significantly greater than the control limb (*p* = 0.005). The intact limb knee muscle CCA was not different than that of the control limb (*p* = 0.105). During late swing the residual limb knee muscle CCA was significantly greater than the intact (*p* = 0.002) and control limb (*p* = 0.003). However, the intact limb knee muscle CCA was not different than the control limb (*p* = 0.409).

**Table 4:** CCA values for the knee muscles during three speeds of gait; Mean (SD)

	**Gait cycle phase**					
	**Early-midstance (0-20 %)**			**Late swing (80–100)%**		
**Speed**	**R**^**a**^	**I**	**C**	**R**	**I**	**C**
Slow	0.78	0.72	0.63	1.4	0.53	0.56
	(0.34)	(0.28)	(0.33)	(1.3)	(0.18)	(0.19)
SSWS	0.95	0.80	0.62	1.0	0.49	0.52
	(0.27)	(0.27)	(0.33)	(0.71)	(0.20)	(0.20)
Fast	0.89	0.76	0.61	1.0	0.50	0.58
	(0.22)	(0.24)	(0.33)	(0.67)	(0.21)	(0.19)

^**a**^ R = Residual, I = Intact, C = Control.

## Discussion

Previous studies have reported differences in knee muscle activation patterns of trans-tibial amputees compared to control subjects, specifically in the amount of co-contraction [7,10,13,14]. However, little has been reported of the activation patterns and amounts of co-contraction in the ankle muscles of the residual limb. The first hypothesis of this study was supported by the observation that ankle muscle co-contractions during early stance (0-10%), mid-late stance (20-60%) and early swing (60-80%) were significantly greater in the residual limb, followed by the intact and control limbs. The second hypothesis was partially supported by the observation that knee muscle co-contractions in the residual limb were significantly different than the control limb (but not the intact limb) during early-midstance (0-20%), and were significantly different than both the control limb and the intact limb during late swing (80-100%). The third hypothesis was not supported; specifically, there was not an effect of walking speed on co-contraction levels for either joint.

Patterns of able-bodied control limb ankle and knee muscle activation were consistent with previous research [1-4,6,10,24,25]. The intact limb ankle and knee muscle activation patterns were also similar to published data [10,26], and demonstrated similar overall patterns compared to the control limb. Residual limb ankle musculature exhibited greater co-contraction than the intact and control limbs. Residual limb knee musculature exhibited more similar patterns to intact and control limbs compared to the ankle musculature; however unusual co-contractions were still present during early stance. The knee muscle co-contraction observations are in agreement with previous studies [7,10,12]. Furthermore, increased residual limb knee co-contractions were observed compared to controls during both early stance and late swing; similar to previously reported results [11].

### Ankle musculature

During early stance (0-10%) the residual limb TA was activated similar to normal levels. In able-bodied gait the MG is ordinarily quiet during this phase; however, in the residual limb it was activated to almost the same level as the TA. During normal gait, from heel-strike to foot-flat the TA contracts eccentrically to provide a dorsiflexor moment about the ankle allowing controlled plantar flexion to foot-flat [6,25]. In the able-bodied control limb, the TA and MG played the expected roles and co-contraction was minimal. In amputee subjects, intact limb co-contraction levels were found to be greater than the control limb. Based on previously published theories this could potentially be occurring to provide a compensatory stabilization effect to absorb extra shock during heel-strike [19,21,27]. The prosthetic limb during this phase is preparing for toe-off and generates a decreased amount of push off power (compared to controls) due to the passive nature of the prosthetic foot resulting in a more abrupt landing for the intact limb [27]. The residual limb demonstrated the largest co-contraction during this phase among all limbs. This may represent a strategy to stabilize the limb system as heel strike and the transition to foot flat occurs. The notion of co-contraction as a stabilization mechanism has been reported previously [9,19]. Plantar flexion from heel strike to foot flat is normally made possible due to the mobility of the human ankle. However, prosthetic devices generally do not facilitate this function as they do not have an eccentrically controlled rotational ankle joint [7,8,26]. This results in the need for increased stability as the amputees have a reduced base of support while on their rear foot for an extended time following heel strike.

During mid-late stance (20-60%), residual limb ankle musculature did not exhibit normal patterns. Similar to the early stance phase of gait, the intact limb exhibited greater co-contraction levels compared to the control limb, but less than the residual limb. This may be due to compensatory strategies employed by the intact limb to provide additional support [9]. Residual limb co-contraction was observed to be greatest during mid-late stance, possibly providing increased stiffness at the stump-socket interface to assist single limb support arising from limited prosthetic foot function [19].

In early swing (60-80%) residual limb activation patterns showed that the MG is activated to almost the same level as the TA. The residual limb co-contraction during this period may be the limb’s attempt to increase suspension as the prosthetic socket undergoes a distraction force from the residual limb during the initiation of swing. Intact limb muscle activation followed patterns similar to control limbs, but exhibited a slightly larger level of co-contraction than control muscles. The reason for this slight increase remains unclear. Future work may confirm this phenomenon and clarify the mechanism underlying this observation.

### Knee musculature

During early-midstance (0-20%) increased residual limb knee muscle co-contraction was observed as BF activation was greater than the control limb. The extra BF activation may be a means of providing co-contraction for additional stability during this phase of prolonged heel-only contact in the early stance portion of gait as described in the discussion above. These findings are in agreement with previous work [7-9,11,12].

During late swing (80-100%) the limb is preparing for heel impact. Muscle activation patterns in control and intact limbs followed anticipated patterns, resulting in co-contraction levels within the expected range. However, residual limb co-contraction was significantly greater than both the control and intact limb (*p <* 0.003). This finding is contrary to previous research which reported equal VL and BF ratios in both residual and intact limbs during swing [11]. In the present study the BF had much lower activation levels in the residual limb, resulting in a high level of apparent co-contraction due to the CCA calculation’s direct comparison. In some subjects this co-contraction may represent an anticipation mechanism as the limb prepares for the impact and inherent instability of heel strike. However, in cases where BF activation is minimal, the calculated co-contraction levels may not fully reflect functional demands due to the nature of the formula. The BF activation in some cases was minimal because the peak was time-shifted and delayed. A delayed peak has been reported previously and is thought to be related to lack of muscle strength [12]. This observation may be confirmed and examined in more detail in future studies of knee and ankle kinetics.

Co-contraction can be quantified using a variety of techniques, however there is no gold standard for quantifying co-contraction levels since each method has inherent limitations. Several of the quantification methods that have been reported can be grouped within four categories [28]. The first category is to use visual estimates of EMG magnitude or percentage overlap in each EMG pair [29,30]; however this method can be influenced by crosstalk and requires normalization that at times may not be possible. The second category of quantification entails normalizing the antagonist EMG to the percent of the maximum voluntary contraction of that same muscle during an agonist contraction [31]. This method is only advantageous when the antagonist is measured under the same circumstances as the agonist and does not consider the antagonist activity or the contribution of the antagonist to the resultant joint moment. The third widely used method involves quantifying the antagonist moment using mathematical modeling that assumes a linear EMG/moment relationship [32,33]. However, the relationship between EMG and muscle force or joint moment is not always considered to be linear. The fourth commonly used method is using a ratio of the EMG activity of the antagonist to agonist [23,34]. While this method can be distorted if the agonist presents minimal recruitment, it is the method used in this study because during gait it was assumed that the agonist should have adequate recruitment to provide movement to the joint. More information about these methods can be found in review articles by Busse et al. [28] and Kellis et al. [35].

It was hypothesized that co-contraction levels would increase with speed demands; however the present findings did not support this hypothesis. It was previously reported that activation generally increases with walking speed, specifically in amputees [1,5,10,20,21]. However, if both muscles in each comparison increase by a similar magnitude the CCA ratio remains constant.

To summarize, amputees appear to co-contract their muscles as a means of enhancing stability and support during specific phases of gait. Due to a lack of prosthetic mobility during early-midstance phases, amputees may employ co-contraction strategies to stabilize and provide extra shock absorption during heel strike and the prolonged phase of rear foot contact. In the mid-late stance phase, co-contractions may be needed to provide assistance and stability during single limb support. In the early swing phase, co-contraction may aid in limb stabilization and provide overall limb stiffness to ensure secure socket suspension. Lastly, in the late swing phase co-contraction strategies may provide an anticipatory stabilizing mechanism for the forthcoming heel strike.

Although the residual and intact limb muscle activation patterns and co-contraction levels differ from that of control limbs, the observed co-contractions may be functionally necessary for stable trans-tibial amputee gait. The results from this study should be taken into consideration and applied to myoelectric controllers in order to accommodate these co-contraction patterns.

There are some inherent limitations in this study. The residual limb was the most variable in activation levels when compared to the intact and control limbs. Increased variation may be due to many factors that were not controlled such as prosthetic device used, residual limb length, muscle re-attachment procedure and time since amputation (longer time since amputation may cause increased residual muscle atrophy). In addition, the walking speed conditions may have been too similar to reveal differences in co-contraction. Future efforts should examine the effect of these factors on residual limb activation and co-contraction patterns. Another limitation is that the present CCA calculation may have overrepresented co-contraction levels in the knee musculature during late swing, where activation levels were low. Future efforts should explore CCA calculations that utilize a relative comparison to prevent the likelihood of singularity.

## Conclusions

The findings of this study have revealed significant limb differences in ankle muscle co-contraction. Additionally, there were significant differences in co-contraction levels of the knee musculature between the residual and control limbs. The occurrence of co-contractions depends on the phase of gait, along with the demands and characteristics of that specific phase. These co-contractions may be a means for enhancing stability during trans-tibial amputee gait and therefore the existence of co-contractions should be considered during the development of future myoelectric controllers.

## Competing interests

The authors declare that they have no competing interests.

## Authors’ contributions

MS collected, processed and analyzed the data and drafted the manuscript. JC and DM contributed to the study design and interpretation of findings. MH conceived of the study, oversaw its design and coordination, contributed to the analysis and interpretation of the findings, and helped to draft the manuscript. All authors read, edited, and approved the final manuscript.
